# Acceptability of long-acting antiretroviral therapy among people living with HIV who use drugs in Vancouver, Canada: A qualitative study

**DOI:** 10.1371/journal.pone.0319010

**Published:** 2025-02-28

**Authors:** Koharu Loulou Chayama, Cara Ng, Isabella Brohman, Manal Mansoor, Will Small, Morgan Philbin, Alexandra B. Collins, Ryan McNeil

**Affiliations:** 1 British Columbia Centre on Substance Use, Vancouver, British Columbia, Canada; 2 Interdisciplinary Studies Graduate Program, University of British Columbia, Vancouver, British Columbia, Canada; 3 Faculty of Health Sciences, Simon Fraser University, Burnaby, British Columbia, Canada; 4 Benioff Homelessness and Housing Initiative, University of California, San Francisco, San Francisco, California, United States of America; 5 School of Medicine, University of California, San Francisco, San Francisco, California, United States of America; 6 Department of Community Health, School of Arts & Sciences, Tufts University, Medford, Massachusetts, United States of America; 7 Department of Internal Medicine, Yale School of Medicine, New Haven, Connecticut, United States of America; 8 Program in Addiction Medicine, Yale School of Medicine, New Haven, Connecticut, United States of America; University of Zimbabwe Faculty of Medicine: University of Zimbabwe College of Health Sciences, ZIMBABWE

## Abstract

**Background:**

People living with HIV (PLHIV) who use drugs face overlapping social-structural inequities that contribute to suboptimal adherence to antiretroviral therapy (ART). Recent research suggests that long-acting antiretroviral therapy (LA-ART) can offer an important alternative to daily oral ART by mitigating adherence barriers, but this work has largely excluded PLHIV who use drugs. We, therefore, explored the acceptability of injectable and transdermal LA-ART among PLHIV who use drugs in Canada, which has no-cost universal access to oral ART. Greater understanding of PLHIV who use drugs’ perspectives on LA-ART will be essential to fully leverage treatment advances and maximize its individual- and population-level benefits.

**Methods:**

From February 2019 to February 2020, we conducted in-depth interviews with 33 PLHIV who use drugs in Vancouver, Canada with suboptimal ART adherence (i.e., ≦  50%). Participants were recruited for interviews through a prospective cohort study. Interviews were audio-recorded, transcribed, and analyzed using inductive and deductive approaches.

**Results:**

Our analysis identified key factors shaping LA-ART acceptability. First, LA-ART enthusiasm was highest among participants who were less satisfied with oral ART regimens due to pill burden or adverse side effects for oral ART and participants who struggled with daily adherence due substance use and social-structural factors (e.g., homelessness, incarceration). Moreover, participants who had physicians they trusted identified their physicians as credible sources of information on LA-ART, and indicated a desire for informed and shared decision-making regarding treatment changes. Finally, participants emphasized that there is no one-size-fits-all treatment approach for PLHIV who use drugs and highlighted the importance of access to a full range of available treatment options, including LA-ART.

**Conclusions:**

There is potential for high LA-ART uptake among PLHIV who use drugs, particularly those who experience adherence barriers due to their substance use and structural vulnerability. While our findings are limited by the specific population and setting, they nevertheless underscore the need for efforts to ensure universal and equitable access to LA-ART.

## Introduction

People living with HIV (PLHIV) are experiencing longer and healthier lives in large part due to advances in antiretroviral therapy (ART) [1–3], including simplified treatment regimens of only one pill per day [4]. ART is effective at suppressing viral load and maintaining CD4 counts, but requires high levels of adherence to achieve optimal treatment response [5]. Even so, the benefits of ART have not been fully realized across all PLHIV. Daily adherence and long-term ART engagement remains a challenge among populations such as PLHIV who use drugs disproportionately impacted by social-structural inequities [6,7]. Adherence to ART is necessary not only to achieve viral suppression and optimal health outcomes among this population, but also to realize the full benefits of treatment as prevention efforts [8].

Adherence to ART among PLHIV who use drugs is constrained by multiple, interrelated factors [9–11]. Research has documented high levels of physical and mental health comorbidities that adversely impact ART adherence, including chronic pain, substance use disorder, and depression [11–13]. Further, research has highlighted how the structural vulnerabilities faced by PLHIV who use drugs can constrain their ART adherence [9–11]. Structural vulnerability is a useful concept to understand suboptimal ART adherence among PLHIV who use drugs [10]. It refers to how the position an individual or a group occupies within a system of power relations based on social (e.g., classism, racism, sexism) and structural inequalities (e.g., poverty, drug criminalization) renders them disproportionately vulnerable to suffering [14]. Even in settings with universal healthcare and coverage of HIV treatment, research has quantitatively and qualitatively documented how constraints rooted in structural vulnerability impact ART adherence and outcomes among PLHIV who use drugs [7,15–21]. For example, incarceration and homelessness contribute to suboptimal ART adherence among PLHIV who use drugs in Vancouver, British Columbia, a setting with no-cost universal access to oral ART [15]. Previous research has called for continued improvements to HIV treatment formulations, accompanied by structural reforms (e.g., provision of truly affordable housing, decriminalization of drugs), to optimize ART adherence among PLHIV who use drugs [10]. As advances in biomedical technologies for HIV treatment are made, acceptability studies among PLHIV who use drugs are needed to ensure equitable uptake and impact of these new technologies.

Long-acting antiretroviral therapy (LA-ART) has begun receiving approval worldwide, including in Australia, Botswana, Canada, the European Union, the United Kingdom, and the United States [22–24]. LA-ART could help address some of the challenges associated with adherence to current treatment regimens [25,26]. Compared to oral ART that must be taken daily, LA-ART is administered on a less-frequent basis (e.g., every 4 or 8 weeks) [25,26]. Different formulations of LA-ART are being explored, including injectable and transdermal (e.g., patches and films) formulations [27,28]. Phase 3 clinical trials have demonstrated non-inferiority of LA-ART compared to oral ART with regard to maintaining viral suppression [25,26]. Importantly, recent observational research from the United States has demonstrated that LA-ART can help people with viremia and challenges with oral ART adherence obtain and maintain viral suppression, including people experiencing homelessness or unstable housing, mental illness, and/or substance use [29]. In addition, an emerging body of research is examining the acceptability of LA-ART [30], including among structurally vulnerable PLHIV such as women living with HIV, sex workers, and people experiencing housing instability and/or substance use [31–33]. A scoping review on the perceptions of long-acting antiretrovirals among key populations of PLHIV identified difficulty maintaining oral ART adherence as one of the most important reasons for preferring LA-ART [30]. As LA-ART is becoming or expected to soon become part of the routine care for PLHIV in many parts of the world, in-depth understanding of the acceptability of LA-ART among PLHIV who are known to disproportionately experience adherence barriers, including PLHIV who use drugs, is critical to the successful implementation of the treatment.

While LA-ART offers promising prospects, little is known about the perspectives of PLHIV who use drugs. To date, no clinical trials have focused exclusively on this population, underscoring the potential risk of people who use drugs from being excluded from available treatment options. One quantitative study found that PLHIV who use drugs were more likely to find LA-ART acceptable than PLHIV who do not use drugs [34]. While only a few studies have qualitatively examined the acceptability of LA-ART among PLHIV who use drugs, these studies have provided important insights, including factors that shape the acceptability of LA-ART among this population [32,33,35,36]. For example, Collins and colleagues found that existing HIV outcomes and experiences with oral ART shaped willingness to consider LA-ART among PLHIV who use drugs in Rhode Island, United States [36]. Research on LA-ART that seeks to understand the perspectives of PLHIV who use drugs is crucial to support the implementation of treatment that is tailored to this population and the social-structural contexts in which they are embedded. However, to our knowledge, no study on the perspectives of PLHIV who use drugs on LA-ART has been conducted in a setting with no-cost universal access to HIV treatment. Understanding perceptions within a universal healthcare setting is critical to assessing factors that influence the acceptability of LA-ART when cost of oral ART is not an impediment, such as in Vancouver. Thus, drawing on the concept of structural vulnerability, we conducted this qualitative study to explore the acceptability of LA-ART among PLHIV who use drugs in Vancouver. Of note, Vancouver provides a unique setting in which to conduct an acceptability study on LA-ART as the province has had longstanding universal coverage of oral ART but restricted coverage of LA-ART based on clinical eligibility criteria (e.g., maintenance of viral suppression ideally for six months or more on oral ART). Greater understanding of the perspectives of PLHIV who use drugs regarding LA-ART will be essential to maximize advances in ART and improve HIV outcomes and quality of life among this population and achieve population benefits of ART-based HIV prevention efforts.

## Methods

### Study design and setting

We draw upon in-depth, semi-structured interviews with 33 PLHIV who use drugs in Vancouver (see Table 1 for demographic information). This study was conducted through an ongoing program of ethno-epidemiological research examining contextual forces that shape HIV prevention and treatment among people who use drugs [9,11,37–40]. Ethno-epidemiological research is a methodological approach in the field of drug research that facilitates deeper insights into how contextual forces influence the acceptability of emerging HIV treatment technologies. The study was approved by the University of British Columbia/Providence Health Care Research Ethics Board.

**Table 1:** Background characteristics of sample (n = 33).

Age	Total	(%)
30–39	3	(9)
40–49	14	(42)
50–59	13	(39)
60–69	3	(9)
Gender		
Man	15	(45)
Two-Spirit	1	(3)
Woman	17	(52)
Race/ethnicity*		
Indigenous	20	(61)
Middle Eastern	2	(12)
White	14	(42)
Drug use in the past 30 days*		
Amphetamine	10	(30)
Cannabis	12	(36)
Cocaine	14	(42)
Crystal methamphetamine	23	(70)
Heroin	18	(55)
Fentanyl	12	(36)
Non-medical prescription opioid use	5	(15)
Other	2	(6)
Years since HIV diagnosis		
Mean (interquartile range)	16.0 (10.5–20.0)	
Years since antiretroviral therapy initiation		
Mean (interquartile range)	12.7 (8.0–18.0)	

*Participants could report multiple categories.

### Study participants

This ethno-epidemiological study was conducted in connection with the AIDS Care Cohort to evaluate Exposure to Survival Services (ACCESS), an open prospective cohort study of PLHIV who use drugs operating in Vancouver’s Downtown Eastside neighbourhood; this is described in greater detail elsewhere [41]. For the ACCESS cohort, individuals who were 18 years of age or older, spoke English, lived in Greater Vancouver, and used illicit drugs other than cannabis in the previous month were recruited through word of mouth, street outreach, and referrals [41]. For the current study, we recruited ACCESS cohort participants who had suboptimal ART adherence rates (i.e., ≦  50%) in the last two semi-annual cohort study follow-up visits, determined through responses to questionnaires completed during cohort study follow-up visits. ACCESS cohort staff invited individuals in the ACCESS cohort who had provided consent to be contacted regarding qualitative studies (approximately 80% of cohort) to participate in the current study through a phone call or during their routine cohort study interview. Individuals who expressed interest were scheduled for an interview with a qualitative interviewer for the current study. Those who could not provide consent for the current study were excluded.

### Data collection

Interviews were conducted at a storefront research office located in Vancouver’s Downtown Eastside neighbourhood. Interviewers explained the study procedures and obtained written informed consent prior to conducting the interviews. The interview guide included questions to facilitate discussion on a range of topics, including: (1) HIV treatment history and perceptions of ART; (2) interaction with care providers regarding HIV treatment; and (3) perceptions of LA-ART, including factors that shape the level of interest in taking LA-ART. Interviews were conducted between February 2019 and February 2020. Interviews averaged 31 minutes (range: 10 - 46 minutes), and were conducted in English, audio recorded, and transcribed verbatim. Participants each received a CAD $30 honorarium following the completion of their interview.

### Data management and analysis

Interview transcripts were imported into NVivo, a qualitative data management and analysis software program, and analyzed using deductive and inductive methods [42]. Research team members reviewed a selection of transcripts and then met as a group to develop the initial coding framework based on preliminary themes emerging from the data and *a priori* themes derived from the interview guide. The data were then coded independently by four members of the research team. During data coding and analysis, the research team met regularly to discuss emerging themes in the data and revise the coding framework. Themes were interpreted through a structural vulnerability lens to emphasize how individual characteristics intersect with social and structural factors to shape acceptability, or lack thereof, towards LA-ART [14]. This was operationalized through discussions of themes and illustrative cases among the research team to better understand the theoretical processes underlying participants’ perspectives on LA-ART.

## Results

### Enthusiasm and willingness to use LA-ART

Many participants expressed enthusiasm and willingness to use both injectable and transdermal formulations of LA-ART. Given that our participants skewed older in age, with most diagnosed with HIV in the 1990s or early 2000s, motivation to adhere to ART was high as they were aware of its health benefits, including improved overall health and lifespan, which they themselves had experienced. Nevertheless, many participants described how adherence to oral ART could be challenging. These participants viewed LA-ART as having the potential to improve treatment adherence by addressing barriers related to current treatment regimens.

#### Addressing pill burden.

Participants felt that LA-ART could make treatment adherence easier by reducing the burden of daily pill administration. This view was particularly prominent among those who took multiple pills to manage their HIV and/or other health conditions. While advances in oral ART have allowed for a single pill regimen, some participants described being on more complex regimens with higher pill counts. This phenomenon has been previously reported in our setting as a result of individuals developing drug resistance to simplified treatment regimens [10]. Moreover, many reported living with comorbidities (e.g., chronic pain, substance use disorder, depression), and the management of these other health conditions also contributed to pill burden. For example, one participant expressed his support for LA-ART as taking multiple pills for his non-HIV health conditions made it difficult to remember to take his oral ART:

*That* [LA-ART] *sounds convenient. Oh, yeah. I would be all for it. I’d be supportive of it because it’s hard to remember to take pills.* […] *Because I take a lot of pills.* [Interviewer: *Oh, for other health conditions?*] *Yeah, like for other things, yeah. For stomach and ulcers, thyroid, all kinds of stuff. I mean, I take a lot of pills. So it’s like it would be nice to get that all at one time per month. That would be really helpful.* (56-year-old Metis man)

#### Side effects.

In addition to simplifying treatment regimens, participants who expressed a strong interest in taking LA-ART often described being less satisfied with oral ART due to side effects they experienced, including nausea, diarrhea, weight loss, abdominal pain, and headaches. One participant described:

*I can’t wait to try it* [LA-ART]*. Yeah, and if it’s something that doesn’t have the side effects that the medication that I’ve been taking do to me now, that would be awesome. I’d be set. I’d be happy. Be happy, happy.* (60-year-old Indigenous woman)

Several participants reiterated these sentiments, including how their side effects necessitated self-management. One participant who got his oral ART co-dispensed with methadone formulation explained how taking these treatments together often made him nauseous and vomit, which he managed by stopping his oral ART:

*So, it was like now you have to do heroin, because you just threw up all the Methadose, right? So that’s why it was the pills that I thought were making me sick, so once I stopped taking the* [ART] *pills, then I wasn’t throwing up anymore.* (64-year-old white man)

#### Socio-structural factors.

Some participants struggled with ART adherence due to the complex realities of their everyday lives and challenges resulting from their structural vulnerability, including housing insecurity. For example, one participant described how becoming unhoused after being evicted, a relatively common experience among PLHIV who use drugs in our setting [17], affected his ability to take his oral ART:

*I did actually for a very short period try the one pill for a very short period […] but I didn’t last longer than three weeks on that because I ended up homeless. So, I went off it right away. I told the doctor, I said, ‘Look, I’m not ready for this because I thought I was, but something happened,’ and I ended up homeless for a stupid reason. It wasn’t even mine. […] I had my rent paid and everything and yet I got kicked out too.* (42-year-old Indigenous woman).

Another participant described how adherence to oral ART was challenging due to the intersection of substance use and structural vulnerabilities, including incarceration. When asked what was going on in his life when he stopped taking his oral ART, he responded:

*I was living in recovery houses, I think, and so there was sporadic using [substance use], but it was more binge using than, you know, on occasion. And I think I was at the jail too there for a while. […] It was a combination of everything, you know. Doing the drugs, being in a bad situation, and not taking the other drug [ART].* (48-year-old white and Indigenous man)

### Desire for shared decision-making

Participants expressed a desire for access to current, evidence-based information on LA-ART, including results from the latest clinical trials. Participants indicated that they wanted to know “*where they did it* [the clinical trial] *and who they did it with, and what the results were, and what the side effects were, and just everything about it*” (64-year-old white man). In particular, participants were interested in receiving information on the mechanism for time release and its effectiveness, as well as side effects of LA-ART, including short and long-term toxicities. Accessing information about these factors were shaped by many participants’ experiences of developing chronic health conditions from previous or current HIV treatment regimens. One participant who attributed her osteoporosis to ART explained:

*The thing with a lot of the new drugs is, they don’t really know too much about the side effects and then later on you end up having that bone disease that old people get [osteoporosis]. I ended up getting that and there’s nothing I could do to sue or anything like that.* (45-year-old white and Middle Eastern woman)

Most participants identified their infectious disease specialists with whom they had established trusting relationships as credible sources of information on LA-ART. Importantly, participants indicated a strong desire for shared decision-making with regards to LA-ART. These desires were often informed by previous experiences of not being involved in decision-making around their treatment. Participants emphasized the importance of effective communication in the decision-making process, including taking time to listen to and understand patients’ needs and concerns. One participant shared:

*If you are a doctor, just make sure you spend a little more time making sure you listen to your patients and make sure you know what they’re taking every day to save it from being spread. Just having an individual, more individual time with your patient to hear their issues.* (53-year-old white woman)

### Access to a full range of available treatment options

Participants interested in LA-ART described how paying out-of-pocket for the treatment would lead them to be less interested, compared to their current treatment which is offered at no cost. One participant (48-year-old white and Indigenous man) described: *“I would stay on the one that I can get publicly funded, of course*.” These participants, many of whom rely on social assistance as their main source of income, explained that they would not be able to afford LA-ART if it were not covered publicly:

*Nobody can afford that kind of money, or Magic Johnson could [Laughs], but he’s exceptional. […] I couldn’t afford it. Even if I could afford it, I don’t think I’d want to spend 80% of my income. […] I’d rather be dead.* (64-year-old white man)

Most participants looked to the public health care system to provide full coverage of the life-saving treatment. When asked whether LA-ART should be publicly-funded, one participant, for example, responded:

*It [LA-AART] should be [publicly] paid for. Fucking right it should be paid for. It should be paid in those countries they have people who are still dying every day from it [HIV]. […] Health care for everybody everywhere. Everywhere. […] Nobody should be fucking dying like a plague. Not anymore.* (43-year-old Indigenous Two Spirit participant)

Participants emphasized that there is no ‘one-size-fits-all’ treatment approach for PLHIV and highlighted the importance of access to a full range of available treatment options, including LA-ART. Throughout the interviews, participants emphasized the need to recognize that individuals are different and respond to treatment in different ways. As one participant described:

*Everybody’s made up differently, so we all react differently to different things. […] So you’d have to try it to figure out if it would work for you or not, right?* (51-year-old white woman)

When asked about their preferences for LA-ART formulations, many participants did not prefer one LA-ART formulation over the other. However, a few participants preferred injectable LA-ART due to concerns over lack of adhesion of transdermal formulations, which they had experienced with other medicated patches (e.g., nicotine and fentanyl patches), and a few participants preferred transdermal formulations due to fear of injections and injection-related infections. Participant accounts underscored the significance of access to a range of LA-ART delivery technologies.

## Discussion

The development of LA-ART marks an important step forward in the treatment of HIV, particularly for PLHIV who experience challenges with ART adherence. Our study contributes to the emerging body of literature on the acceptability of LA-ART among structurally vulnerable PLHIV. Specifically, this study adds the perspectives of PLHIV who use drugs, a population that continues to experience suboptimal adherence to ART and lower rates of viral suppression. Our analysis of participant accounts identified key factors shaping LA-ART acceptability. Participants who were less satisfied with oral ART regimens expressed enthusiasm and willingness to use LA-ART. Participants identified physicians with whom they had trusting relationships as credible sources of information on LA-ART and indicated a strong desire for informed and shared decision-making with regards to ART. Participant narratives demonstrated that there is no one-size-fits-all treatment approach for PLHIV who use drugs and highlighted the importance of access to a full range of available treatment options, including LA-ART.

Our findings suggest that PLHIV who use drugs could be particularly well-positioned to benefit from LA-ART. Participants who expressed a strong interest in taking LA-ART were often less satisfied with their current treatment regimens or had struggled with daily adherence. Our findings are in-line with previous research on LA-ART suggesting that it could alleviate the burden of daily pill administration among structurally vulnerable PLHIV [31,33,43]. We extend the existing literature by identifying that LA-ART could help to alleviate the burden associated with oral ART among people impacted by polypharmacy (i.e., five or more medications) [44], such as those living with comorbidities. LA-ART may serve as an important treatment option, particularly as PLHIV who use drugs are getting older and living with multiple comorbidities [11].

Moreover, our findings highlight the potential of LA-ART to benefit PLHIV who use drugs impacted by structural vulnerabilities that interfere with ART adherence (e.g., housing insecurity, incarceration). This aligns with findings from a recent study by Fletcher and colleagues, which found that PLHIV experiencing housing instability and/or substance use preferred LA-ART over a daily pill regimen as it addressed conditions unique to their structural vulnerability (e.g., not having to worry about their medications being stolen) [33]. Alongside structural reforms such as the provision of truly affordable housing and decriminalization of drugs that address the underlying social, economic, and political factors that shape ART adherence, LA-ART offers a critical opportunity to impact HIV outcomes and quality of life among PLHIV who use drugs. Optimizing ART adherence among structurally vulnerable PLHIV, including people who use drugs and other key populations identified by UNAIDS, will accelerate progress towards ending AIDS by 2030.

Our findings underscore the importance of shared decision-making with regards to LA-ART. Shared decision-making is particularly appropriate in cases where there is not a single ‘best’ treatment option [45], such as with HIV treatment. Previous studies have identified that PLHIV value involvement in decision-making regarding HIV treatment [46,47], and prefer levels of involvement that may be higher than those of other groups [48]. This enhanced desire for involvement in decision-making may be attributed to the fact that HIV treatments have come not only with benefits but also with undesirable side effects, including long-term toxicities [48]. Moreover, research has suggested that satisfaction with the amount of personal control over treatment decisions is important for ART uptake among PLHIV [49]. Shared decision-making that respects patient preferences should be adopted to ensure that treatment plans optimize the health and wellbeing of PLHIV who use drugs.

Finally, our findings highlight the need for British Columbia to expand access to LA-ART. As of March 2024, British Columbia was the only province in Canada without widely accessible LA-ART [50]. While PLHIV in British Columbia have access to oral ART at no cost through the Drug Treatment Program funded by the provincial government, the program currently restricts access to LA-ART for many PLHIV through its clinical eligibility criteria [50,51]. Participant narratives highlighted how cost could be a major barrier to LA-ART uptake among PLHIV who use drugs. This adds to growing calls locally and globally, including by UNAIDS and other global health agencies [52], for immediate and expanded access to LA-ART.

This study has limitations. First, given that our participants were all English-speaking and recruited through a longstanding cohort study, our sample population may be over-representative of individuals who are well-connected to health and social services. Therefore, their experiences may not reflect those of individuals who are disconnected from health and social services and among the most structurally vulnerable, such as new immigrants. Second, many of our participants have experiences with older HIV treatment regimens as they were diagnosed with HIV in the 1990s or early 2000s. Thus, their experiences may not reflect those of individuals who were diagnosed with HIV more recently, or younger PLHIV. Finally, our study was undertaken in a setting with a longstanding universal coverage of ART and other treatment supports for PLHIV. Research conducted in settings without these supports may uncover additional treatment-related experiences. Nevertheless, this study offers in-depth insights into the perspectives on LA-ART among a population underrepresented in literature.

## Conclusions

LA-ART has potential for high uptake among PLHIV who use drugs, particularly those who experience barriers to adherence to current oral ARV treatment regimens due to their substance use and structural vulnerability. Our findings underscore the need for efforts to ensure universal and equitable access to LA-ART, including interventions to improve shared decision-making and quality of care.

## References

[1] Hogg RS, O’Shaughnessy MV, Gataric N, Yip B, Craib K, Schechter MT, et al. Decline in deaths from AIDS due to new antiretrovirals. Lancet. 1997;349(9061):1294. doi: 10.1016/S0140-6736(05)62505-6 9142067

[2] Trickey A, Zhang L, Sabin CA, Sterne JAC. Life expectancy of people with HIV on long-term antiretroviral therapy in Europe and North America: a cohort study. Lancet Healthy Longev. 2022;3:S2. doi: 10.1016/s2666-7568(22)00063-0

[3] Antiretroviral Therapy Cohort Collaboration. Survival of HIV-positive patients starting antiretroviral therapy between 1996 and 2013: a collaborative analysis of cohort studies. Lancet HIV. 2017;4(8):e349–56. doi: 10.1016/S2352-3018(17)30066-8 28501495 PMC5555438

[4] Sutton SS, Hardin JW, Bramley TJ, D’Souza AO, Bennett CL. Single- versus multiple-tablet HIV regimens: adherence and hospitalization risks. Am J Manag Care. 2016;22(4):242–8. 27143289

[5] Bezabhe WM, Chalmers L, Bereznicki LR, Peterson GM. Adherence to antiretroviral therapy and virologic failure: a meta-analysis. Medicine (Baltimore). 2016;95(15):e3361. doi: 10.1097/MD.0000000000003361 27082595 PMC4839839

[6] Malta M, Strathdee SA, Magnanini MMF, Bastos FI. Adherence to antiretroviral therapy for human immunodeficiency virus/acquired immune deficiency syndrome among drug users: a systematic review. Addiction. 2008;103(8):1242–57. doi: 10.1111/j.1360-0443.2008.02269.x 18855813

[7] Bazzi AR, Drainoni M-L, Biancarelli DL, Hartman JJ, Mimiaga MJ, Mayer KH, et al. Systematic review of HIV treatment adherence research among people who inject drugs in the United States and Canada: evidence to inform pre-exposure prophylaxis (PrEP) adherence interventions. BMC Public Health. 2019;19(1):31. doi: 10.1186/s12889-018-6314-8 30621657 PMC6323713

[8] Saag MS, Benson CA, Gandhi RT, Hoy JF, Landovitz RJ, Mugavero MJ, et al. Antiretroviral drugs for treatment and prevention of HIV infection in adults: 2018 recommendations of the International Antiviral Society–USA panel. JAMA. 2018;320(4):379. 30043070 10.1001/jama.2018.8431 PMC6415748

[9] Fleming T, Collins AB, Bardwell G, Fowler A, Boyd J, Milloy MJ, et al. A qualitative investigation of HIV treatment dispensing models and impacts on adherence among people living with HIV who use drugs. PLoS One. 2021;16(2):e0246999. doi: 10.1371/journal.pone.0246999 33635886 PMC7909635

[10] McNeil R, Kerr T, Coleman B, Maher L, Milloy MJ, Small W. Antiretroviral therapy interruption among HIV positive people who use drugs in a setting with a community-wide HIV treatment-as-prevention initiative. AIDS Behav. 2017;21(2):402–9. doi: 10.1007/s10461-016-1470-2 27351192 PMC5360157

[11] Chayama KL, Ng C, Small W, Ivsins A, McNeil R. “It’s a burden, it’s a nuisance. I wish I didn’t have these other ailments”: a qualitative exploration of comorbidities management among older people living with HIV who use drugs in Vancouver, British Columbia. J Int AIDS Soc. 2021;24(10):e25785. doi: 10.1002/jia2.25785 34636148 PMC8505831

[12] Denis C, Morales K, Wu Q, Metzger D, Cheatle M. Association between diagnoses of chronic noncancer pain, substance use disorder, and HIV-related outcomes in people living with HIV. JAIDS J Acquir Immune Defic Syndr. 2019;82(2):S142–7. 31658202 10.1097/QAI.0000000000002179 PMC6822377

[13] Uthman OA, Magidson JF, Safren SA, Nachega JB. Depression and adherence to antiretroviral therapy in low-, middle- and high-income countries: a systematic review and meta-analysis. Curr HIV/AIDS Rep. 2014;11(3):291–307. doi: 10.1007/s11904-014-0220-1 25038748 PMC4359613

[14] Quesada J, Hart LK, Bourgois P. Structural vulnerability and health: Latino migrant laborers in the United States. Med Anthropol. 2011;30(4):339–62. doi: 10.1080/01459740.2011.576725 21777121 PMC3146033

[15] Joseph B, Kerr T, Puskas CM, Montaner J, Wood E, Milloy M-J. Factors linked to transitions in adherence to antiretroviral therapy among HIV-infected illicit drug users in a Canadian setting. AIDS Care. 2015;27(9):1128–36. doi: 10.1080/09540121.2015.1032205 25915438 PMC4830351

[16] Socias ME, Milloy M-J. Substance use and adherence to antiretroviral therapy: what is known and what is unknown. Curr Infect Dis Rep. 2018;20(9):36. doi: 10.1007/s11908-018-0636-7 30066113 PMC6375705

[17] Kennedy MC, Kerr T, McNeil R, Parashar S, Montaner J, Wood E, et al. Residential eviction and risk of detectable plasma HIV-1 RNA viral load among HIV-positive people who use drugs. AIDS Behav. 2017;21(3):678–87. doi: 10.1007/s10461-016-1315-z 26906022 PMC4995155

[18] Ickowicz S, Salleh NAM, Fairbairn N, Richardson L, Small W, Milloy M-J. Criminal justice system involvement as a risk factor for detectable plasma HIV viral load in people who use illicit drugs: a longitudinal cohort study. AIDS Behav. 2019;23(9):2634–9. doi: 10.1007/s10461-019-02547-z 31236749 PMC6773261

[19] Milloy M-J, Kerr T, Buxton J, Rhodes T, Krusi A, Guillemi S, et al. Social and environmental predictors of plasma HIV RNA rebound among injection drug users treated with antiretroviral therapy. J Acquir Immune Defic Syndr. 2012;59(4):393–9. doi: 10.1097/QAI.0b013e3182433288 22134149 PMC3299888

[20] Richardson LA, Kerr TH, Dobrer S, Puskas CM, Guillemi SA, Montaner JSG, et al. Socioeconomic marginalization and plasma HIV-1 RNA nondetectability among individuals who use illicit drugs in a Canadian setting. AIDS. 2015;29(18):2487–95. doi: 10.1097/QAD.0000000000000853 26558546 PMC4646709

[21] Small W, Milloy MJ, McNeil R, Maher L, Kerr T. Plasma HIV-1 RNA viral load rebound among people who inject drugs receiving antiretroviral therapy (ART) in a Canadian setting: an ethno-epidemiological study. AIDS Res Ther. 2016;13:26. doi: 10.1186/s12981-016-0108-9 27462360 PMC4960678

[22] Thoueille P, Choong E, Cavassini M, Buclin T, Decosterd LA. Long-acting antiretrovirals: a new era for the management and prevention of HIV infection. J Antimicrob Chemother. 2022;77(2):290–302. doi: 10.1093/jac/dkab324 34499731 PMC8809192

[23] Delany-Moretlwe S, Flexner C, Bauermeister JA. Advancing use of long-acting and extended delivery HIV prevention and treatment regimens. J Int AIDS Soc. 2023;26(Suppl 2):e26126. doi: 10.1002/jia2.26126 37439079 PMC10338994

[24] Norcross C, Ombajo LA, Kassim S, Garrett N, Cresswell FV, Ruzagira E. Long-acting antiretrovirals: research and implementation considerations in Africa. Lancet HIV. 2023;10(7):e428–9. doi: 10.1016/S2352-3018(23)00134-0 37419566

[25] Swindells S, Andrade-Villanueva J, Richmond G, Rizzardini G, Baumgarten A, Masiá M. Long-acting cabotegravir and rilpivirine for maintenance of HIV-1 suppression. N Engl J Med. 2020;382(12):1112–23. 32130809 10.1056/NEJMoa1904398

[26] Orkin C, Arasteh K, Górgolas Hernández-Mora H, Pokrovsky V, Overton E, Girard P. Long-acting cabotegravir and rilpivirine after oral induction for HIV-1 infection. N Engl J Med. 2020;382(12):1124–35. 32130806 10.1056/NEJMoa1909512

[27] Limenh LW. Advances in the transdermal delivery of antiretroviral drugs. SAGE Open Med. 2024;12:20503121231223600. doi: 10.1177/20503121231223600 38249942 PMC10798114

[28] Cobb DA, Smith NA, Edagwa BJ, McMillan JM. Long-acting approaches for delivery of antiretroviral drugs for prevention and treatment of HIV: a review of recent research. Expert Opin Drug Deliv. 2020;17(9):1227–38. doi: 10.1080/17425247.2020.1783233 32552187 PMC7442675

[29] Gandhi M, Hickey M, Imbert E, Grochowski J, Mayorga-Munoz F, Szumowski JD, et al. Demonstration project of long-acting antiretroviral therapy in a diverse population of people with HIV. Ann Intern Med. 2023;176(7):969–74. doi: 10.7326/M23-0788 37399555 PMC10771861

[30] Sued O, Nardi N, Spadaccini L. Key population perceptions and opinions about long-acting antiretrovirals for prevention and treatment: a scoping review. Curr Opin HIV AIDS. 2022;17(3):145–61. doi: 10.1097/COH.0000000000000734 35439789

[31] Kerrigan D, Sanchez Karver T, Muraleetharan O, Savage V, Mbwambo J, Donastorg Y, et al. “A dream come true”: perspectives on long-acting injectable antiretroviral therapy among female sex workers living with HIV from the Dominican Republic and Tanzania. PLoS One. 2020;15(6):e0234666. doi: 10.1371/journal.pone.0234666 32530939 PMC7292359

[32] Philbin MM, Parish C, Bergen S, Kerrigan D, Kinnard EN, Reed SE, et al. A qualitative exploration of women’s interest in long-acting injectable antiretroviral therapy across six cities in the women’s interagency HIV study: intersections with current and past injectable medication and substance use. AIDS Patient Care STDS. 2021;35(1):23–30. doi: 10.1089/apc.2020.0164 33400587 PMC7826427

[33] Fletcher L, Burrowes SAB, Khan GK, Sabin L, Johnson S, Kimmel SD, et al. Perspectives on long-acting injectable HIV antiretroviral therapy at an alternative care site: a qualitative study of people with HIV experiencing substance use and/or housing instability. Harm Reduct J. 2023;20(1):4. doi: 10.1186/s12954-023-00730-z 36627679 PMC9830853

[34] Williams J, Sayles HR, Meza JL, Sayre P, Sandkovsky U, Gendelman HE, et al. Long-acting parenteral nanoformulated antiretroviral therapy: interest and attitudes of HIV-infected patients. Nanomedicine (Lond). 2013;8(11):1807–13. doi: 10.2217/nnm.12.214 23611617 PMC3987826

[35] Rutstein SE, Sibley AL, Huffstetler HE, Nguyen TTD, Tran HV, Le Minh G, et al. Acceptability and feasibility of long-acting injectable antiretroviral therapy for HIV-infected persons who inject drugs in Vietnam: a qualitative study. Lancet Reg Health West Pac. 2022;31:100603. doi: 10.1016/j.lanwpc.2022.100603 36879789 PMC9985034

[36] Collins AB, Macon EC, Langdon K, Joseph R, Thomas A, Dogon C, et al. Perceptions of long-acting injectable antiretroviral therapy among people living with HIV who use drugs and service providers: a qualitative analysis in Rhode Island. J Urban Health. 2023;100(5):1062–73. doi: 10.1007/s11524-023-00755-6 37563518 PMC10618145

[37] Chayama K, Valleriani J, Ng C, Haines-Saah R, Capler R, Milloy MJ, Small W, McNeil R. The role of cannabis in pain management among people living with HIV who use drugs: a qualitative study. Drug Alcohol Rev. 2021. 10.1111/dar.13294 PMC8580359 33843074

[38] Chayama K, Ng C, Fleming T, Small W, Sue K, McNeil R. Housing-based syringe services programs to improve access to safer injecting equipment for people who inject drugs in Vancouver, Canada: a spatially oriented qualitative study. Harm Reduct J. 2023;20(1):126. 37679789 10.1186/s12954-023-00862-2 PMC10483728

[39] Fleming T, Collins AB, Bardwell G, Fowler A, Boyd J, Small W, McNeil R. Home and health among people living with HIV who use drugs: a qualitative study. Int J Drug Policy. 2020;80:102729. doi: 10.1016/j.drugpo.2020.102729 32388481 PMC8023234

[40] Fleming T, Valleriani J, Ng C, Maher L, Small W, McNeil R. Acceptability of a hypothetical preventative HIV vaccine among people who use drugs in Vancouver, Canada. BMC Public Health. 2020;20(1):1081. 32646390 10.1186/s12889-020-09202-6 PMC7350753

[41] Strathdee SA, Patrick DM, Currie SL, Cornelisse PG, Rekart ML, Montaner JS, et al. Needle exchange is not enough: lessons from the Vancouver injecting drug use study. AIDS. 1997;11(8):F59–65. doi: 10.1097/00002030-199708000-00001 9223727

[42] Creswell J. Research design: qualitative, quantitative, and mixed methods approaches. 3rd ed. Washington, D.C.: Sage; 2009.

[43] Philbin M, Parish C, Kinnard E, Reed S, Kerrigan D, Alcaide M. Multisite study of women living with HIV’s perceived barriers to, and interest in, long-acting injectable antiretroviral therapy. J Acquir Immune Defic Syndr. 2020;84(3):263–70. 32530905 10.1097/QAI.0000000000002337 PMC7483266

[44] Varghese D, Ishida C, Haseer Koya H. Polypharmacy. In: StatPearls [Internet]. Treasure Island (FL): StatPearls Publishing; 2024 [cited 2024 Mar 28]. Available from: http://www.ncbi.nlm.nih.gov/books/NBK532953/30422548

[45] Stiggelbout AM, Weijden TVD, Wit MPTD, Frosch D, Legare F, Montori VM. Shared decision making: really putting patients at the centre of healthcare. BMJ. 2012;344(1):e256. 22286508 10.1136/bmj.e256

[46] Marelich WD, Johnston Roberts K, Murphy DA, Callari T. HIV/AIDS patient involvement in antiretroviral treatment decisions. AIDS Care. 2002;14(1):17–26. doi: 10.1080/09540120220097900 11798402

[47] Gerbert B, Love C, Caspers N, Linkins K, Burack JH. “Making all the difference in the world”: how physicians can help HIV-seropositive patients become more involved in their healthcare. AIDS Patient Care STDS. 1999;13(1):29–39. doi: 10.1089/apc.1999.13.29 11362084

[48] Beach MC, Duggan PS, Moore RD. Is patients’ preferred involvement in health decisions related to outcomes for patients with HIV? J Gen Intern Med. 2007;22(8):1119–24. doi: 10.1007/s11606-007-0241-1 17514382 PMC2305727

[49] Cooper V, Buick D, Horne R, Lambert N, Gellaitry G, Leake H, et al. Perceptions of HAART among gay men who declined a treatment offer: preliminary results from an interview-based study. AIDS Care. 2002;14(3):319–28. doi: 10.1080/09540120220123694 12042077

[50] Thayaparan A. HIV advocates frustrated over access to monthly treatment, but researcher cautions over eligibility. CBC News [Internet]. 2023. Available from: https://www.cbc.ca/news/canada/british-columbia/bc-hiv-injection-1.6801265

[51] BC Centre for Excellence in HIV/AIDS. RE: Cabotegravir and Rilpivirine extended-release injectable suspensions [Internet]. 2023. Available from: https://www.bccfe.ca/sites/default/files/uploads/dear-doctor-letters/cabenuva_cfe_2022-09-22.pdf

[52] UNAIDS J. Delays in global, affordable access to long-acting, injectable HIV medicines would cost lives, say AIDS campaigners [Internet]. UNAIDS; 2022. Available from: https://www.unaids.org/en/resources/presscentre/featurestories/2022/november/20221116_long-acting-injectable-HIV-medicines

